# Physical activity counselling by physicians - Results from the KomPaS study

**DOI:** 10.25646/7148

**Published:** 2021-06-16

**Authors:** Susanne Jordan, Anne Starker

**Affiliations:** Robert Koch Institute, Berlin, Department of Epidemiology and Health Monitoring

**Keywords:** PROMOTION OF PHYSICAL ACTIVITY, BEHAVIOUR-RELATED PREVENTION, PHYSICIAN’S RECOMMENDATIONS, PHYSICAL ACTIVITY

## Abstract

Physical activity counselling aimed at promoting physical and sporting activity is easily accessible and has the potential to reach many people. Until now, very little has been known about the factors influencing physical activity counselling and their frequency. However, the study ‘KomPaS: survey on communication and patient-safety’, provides current data about this topic. The analyses published here are based on data from 4,561 people aged 18 or older who were interviewed by telephone between May and September 2017 and who stated that they had visited a physician’s practice or outpatient clinic in the last twelve months. 28.6% of participants reported having received a physician’s counselling about sporting activity during the past twelve months. Sex, age and socioeconomic status have an impact on how frequently participants reported a physical activity counselling by a physician as well as changes to physical activity. As such, differences associated with sex, age and socioeconomic status should be taken into account during physical activity counselling so as to provide various population groups with targeted support.

## Introduction

Physical activity can help reduce the risk of noncommunicable diseases and counteracts the aggravation of chronic diseases [1, 2]. In Germany, less than half of the adult population meets the World Health Organization’s recommendations on physical activity [3]. As such, the promotion of physical and sporting activity remains one of the central challenges faced by health promotion and disease prevention in Germany today. A wide variety of measures are currently used to face these challenges, and, in addition to environmental and policy-related approaches and measures that target people’s daily lives, this includes the provision of physician’s counselling within health care settings [4].

As many people visit a physician’s practice at least once a year [5, 6], and most people still tend to consult physicians about health-related issues [7], physical activity counselling can be used to provide patients highly accessible, needs-based advice on physical activity. Furthermore, assessments of physical activity can also be used to deliver tailored counselling to patients, which should include referral to experts on physical activity, sports clubs and other providers of physical and sporting activity [8].

In Germany, the 2015 Preventive Health Care Act strengthened physician’s counselling such as that medical health checks can include prevention-oriented counselling, such as advice about physical activity. The Act also allows that physicians can issue patients with a letter recommending individual behaviour-related preventive measures offered by their health insurance [9]. This ties in with experiences made in nine federal states in Germany, where physicians have been able to prescribe patients with preventive services that promote physical activity [10].

Until now, there have been very few studies about the frequency of physician’s physical activity counselling and their influencing factors, particularly at the population level. Nevertheless, data are available from the German National Health Interview and Examination Survey 1998 (GNHIES98) and the German Health Interview and Examination Survey for Adults (DEGS1). In 1998, about one tenth of the population aged between 18 and 64 reported having attended a physical activity counselling. These figures decreased from 9.3% to 7.7% among women and from 11.1% to 9.4% among men in the period between the studies (1997–1999 and 2008–2011) [11, 12]. The study ‘KomPaS: survey on communication and patient-safety’ provides current data about the frequency of physician’s counselling on physical and sporting activity from the point of view of the population. This section of the study focused on the extent to which uptake of physical activity counselling differs according to sex, age and socioeconomic status.

**KomPaS study**

KomPaS: survey on communication and patient-safety

**Data holder:** Robert Koch Institute

**Objectives:** Describe informational needs, health literacy, patient safety, informed decision-making and physician’s counselling from the population’s point of view as part of patients’ information, decision-making and communication-related behaviour and the doctor-patient relationship.

**Survey method:** Computer-assisted telephone interview survey

**Study design:** Cross-sectional study

**Population:** German-speaking resident population in private households in Germany aged 18 or over

**Sampling:** Telephone sample comprising 60% landline and 40% mobile phone numbers

**Survey period:** May to September 2017

**Response rate:** 17,2%

**Sample size:** 5,053 participants

## Indicator

Data on the use of physical activity counselling provided by physicians was collected for the KomPaS study using a representative telephone survey undertaken between May and September 2017. The survey covered the adult resident population in Germany. Participants were asked whether they had visited a physician’s practice or an outpatient clinic in the past twelve months. Those who answered in the affirmative were then asked: ‘Were you provided with counselling about any of the following health-related topics during any of these visits in the last 12 months?’. The topics covered physical activity but also nutrition and stress management. Participants who reported a counselling were asked whether they believed the counselling had led them to change their behaviour (‘Did you modify your behaviour as a result’, response categories: ‘yes’ and ‘no’). These items were taken from the DEGS1 study [13] and adapted from a written survey for use with a telephone survey.

The following analyses are based on data from 4,561 people aged 18 or over (2,636 women, 1,925 men) who visited a physician’s practice or outpatient clinic in the twelve months prior to the KomPaS study, which was the case with 90.8% of women and 85.6% of men. This article reports relative frequencies with 95% confidence intervals (95% CI) stratified by sex, age and socioeconomic status. Wide confidence intervals indicate a greater level of statistical uncertainty in the results. A significant difference is assumed in cases where the p-value is less than 0.05 after taking weighting and survey design into account. In order to provide representative results for the total resident population in Germany, the household sizes in the sample were adjusted to reflect the distribution in the population. This was followed by design and adjustment weighting to correct for deviations from the population structure (as of 31 December 2016) with regard to age, sex, education and place of residence (federal state). All analyses were carried out using Stata 15.1 [14]. A detailed description of the methodology and the sample used for the KomPaS study can be found in the study report [15].

## Results and discussion

Almost one third of participants (28.6%) reported that they had attended a physical activity counselling provided by a physician on sporting activity during the past twelve months (Table 1). No significant differences were identified between the sexes, and relative frequencies differed only slightly (women 27.4%, men 29.9%). The proportion of women who reported a counselling did not change significantly with age. In contrast, 45- to 64-year-old men reported a counselling much more frequently than men in other age groups. For example, 45- to 64-year-olds differed from the 30- to 44-year-old group by 11.2 percentage points, a frequency that is almost one third higher. Although no significant differences were identified for socioeconomic status within groups of women or men, differences were identified between the sexes: 34.6% of men in the high socioeconomic status group reported having attended a counselling provided by a physician on sporting activity, compared to 23.3% of women in the same status group.

According to data from the KomPaS study from 2017, the frequency of physical activity counselling by physicians has more than doubled since DEGS1 (2008–2011), when around one tenth of those surveyed reported having attended a counselling about sporting activity [11, 12]. Even if the two surveys used different survey modes (a written questionnaire versus a telephone-based interview), they asked the same questions, albeit adapted to the mode in question, and the results are therefore comparable. Reasons for the higher frequency are likely to lie in the increased focus on physical activity in health promotion, prevention and therapy over the last decade, which is also reflected in measures such as the ‘prescription of physical activity’ [10] and the introduction of the Preventive Health Care Act in 2015 with its physician’s recommendations on prevention. The 2019 Prevention Report by Germany’s National Prevention Conference states that initial, non-representative analyses also indicate that physicians most frequently prescribed physical activity programmes when issuing a prevention recommendation [9]. Further research should clarify the reason why 45- to 64-year-old men and men in the high socioeconomic status group most frequently reported physical activity counselling. This is important because it had been assumed that the low socioeconomic status group would display the highest frequency of counselling because people in this group generally do sports less often and face greater health burdens than people in other status groups [16, 17].

**Figure 1:**
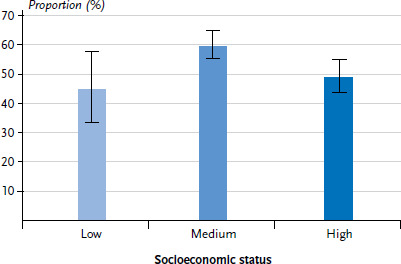
Self-reported changes in behaviour after a physician’s counselling about physical and sporting activity by socioeconomic status (n=1,343) Source: KomPaS study (2017)

When participants were asked whether they had changed their behaviour due to a physician’s counselling about sporting activity, more than half of women and men (total: 55.6%) stated that they had done so. Due to the low number of cases, no sex-specific results are reported here for age or socioeconomic status. In general, no significant differences were identified by age but significant differences were found between the medium and high socioeconomic status group. Participants in the medium status group stated significantly more frequently that they had changed their behaviour after a physician’s counselling than the high status group (60.1% versus 49.1%, Figure 1). The differences between the medium and the low socioeconomic status group are not statistically significant. Further analyses should investigate the reasons for the differences in socioeconomic status and sex in the implementation of physical activity counselling.

It is important to note that as the KomPaS study is a cross-sectional study, no causal conclusions can be drawn from the results presented here. Furthermore, the study only collected (self-reported) data on the population’s point of view, and not on the type, quality and impact of physician’s counselling. High-quality individual studies are still lacking, particularly when it comes to the effectiveness of counselling [4]. An analysis using data from DEGS1 showed that participants who reported a counselling were 2.5 times more likely to take part in behavioural preventive measures aimed at promoting physical activity [18]. Overall, however, there is insufficient and contradictory evidence about the effectiveness of physical activity counselling [4]. About half of available studies identify minor short- or medium-term effects [4, 19]. However, the counselling under study often took place within the context of physical activity programmes instead of being individual measures [4]. In addition, the results of a study on the ‘prescription of physical activity’ [20] indicate that physicians need even more information about the importance of physical and sporting activity for health, as well as about the availability of physical activity programmes in their local area. In another study, half of patients surveyed expressed a desire for more support from their health insurers to enable them to take up physical activity [21]. Therefore, further research is needed into patients’ assessments, and at the same time those of the physicians providing counselling, as well as characteristics of the counselling for physical-sporty activity, and, above all, their effectiveness.

The differences highlighted by the KomPaS study in terms of physical activity counselling by sex, age and socioeconomic status indicate that counselling have further potential. In addition to settings-based preventive measures, for example, in companies and the community, counselling by physicians is easily accessible and is able to reach a relatively large section of the population. Structured counselling [22] and successful doctor-patient communication are promising approaches that should be implemented and further strengthened as part of physician’s training [23]. If physical activity counselling by physicians are to be effective, however, the differences associated with sex, age and socioeconomic status need to be taken into account – even if further research is still needed on this issue.

## Key statements

- Almost one third of participants reported having attended a physician’s counselling about sporting activity in the last twelve months.
- Significant differences between the sexes were only identified for the high socioeconomic status group.
- Men in the 45- to 64-year-old age group more frequently reported having attended a counselling than men in other age groups.
- The medium socioeconomic status group more frequently reported having changed their behaviour after a physician’s counselling than the lower or higher status group.

## Figures and Tables

**Table 1:** Frequency of physician’s counselling about physical and sporting activity by sex, age and socioeconomic status (n=2,636 women, n=1,925 men) Source: KomPaS study (2017)

	%	(95% CI)
**Women (total)**	**27.4**	**(25.0-29.8)**
**Age group** 18-29 years 30-44 years 45-64 years ≥65 years	26.7 25.4 29.2 27.0	(18.2-37.4) (20.4-31.1) (25.8-32.7) (23.5-30.8)
**Socioeconomic status** Low Medium High	28.5 27.9 23.3	(21.1-37.3) (25.0-31.0) (20.0-27.1)
**Men (total)**	**29.9**	**(27.3-32.7)**
**Age group** 18–29 years 30–44 years 45–64 years ≥65 years	28.2 23.9 35.1 28.6	(20.6–37.3) (18.1–30.9) (31.1–39.3) (24.6–32.8)
**Socioeconomic status** Low Medium High	30.6 27.2 34.6	(22.0–40.8) (23.8–31.0) (30.6–38.7)
**Total (women and men)**	**28.6**	**(26.8-30.4)**

CI = confidence interval
